# Successful high dose antipsychotic treatment with cariprazine in patients on the schizophrenia spectrum: Real-world evidence from a Spanish hospital setting

**DOI:** 10.3389/fpsyt.2023.1112697

**Published:** 2023-02-24

**Authors:** Lluis Niell Galmes, Elmars Rancans

**Affiliations:** ^1^Infanta Sofia University Hospital, Madrid, Spain; ^2^Department of Psychiatry and Addiction Disorders, Riga Stradins University, Riga, Latvia

**Keywords:** cariprazine, schizophrenia spectrum, real-world evidence, antipsychotic, partial agonist, high dose antipsychotic treatment

## Abstract

Real-world evidence fills in an important gap by providing data on the effectiveness and tolerability of new medications in everyday patients. In this data collection form a Spanish hospital, the effectiveness and tolerability of cariprazine were evaluated in 14 patients who were admitted to the hospital due to an acute episode of schizophrenia or schizoaffective disorder. The collected data included demographic characteristics, history of disorder and previous treatment, and details of cariprazine therapy such as dosing, side effects and measurements of effectiveness *via* scales. Difference between admission and discharge on the Brief Psychiatric Rating Scale (BPRS) and Clinical Global Impression-Severity (CGI-S) scale scores were evaluated using the Wilcoxon Signed-Rank test. Significant improvement was detected in nearly all patients (one patient dropped out) as measured by the BPRS Total, Negative symptom, Positive symptom, and Hostility scores. At admission, patients were markedly-moderately ill and at discharge the severity was reduced to borderline ill and normal according to the CGI-S. The CGI-Improvement scale also indicated very much and much improvement at discharge. Importantly, patients left the hospital with high doses of cariprazine, i.e., 7.5 mg/day or even 9.0 mg/day, but this did not cause safety problems; cariprazine well-tolerated as only a few patients experienced side effects such as akathisia. The results provide novel evidence regarding the tolerability and effectiveness of cariprazine in high doses patients on the schizophrenia spectrum.

## Introduction

Disorders on the schizophrenia spectrum are chronic psychiatric conditions characterized by considerable distortions of thinking and perception driven by three core symptom domains: positive symptoms, negative symptoms and cognitive symptoms (1). Positive symptoms include delusions, hallucinations and disorganized thinking, while negative symptoms can be described by the so-called “5As”: anhedonia, avolition, asociality, blunted affect and alogia (1, 2). Besides the positive and negative symptom manifestations of the schizophrenia spectrum, cognitive symptoms, e.g., problems with thinking and memory, are also prevalent (1). Reflecting to the notion that the boundaries between psychiatric disorders can be blurry, in the 5th edition of the Diagnostic and Statistical Manual of Mental Disorders (DSM-V) the word “spectrum” was included in the description of schizophrenia related disorders (1, 3–5). Currently, schizophrenia spectrum disorders include schizophrenia, schizoaffective disorder, and brief psychotic disorder among others (1).

Cariprazine is a third-generation antipsychotic medication approved for the treatment of schizophrenia in adults by the Food and Drug Administration (FDA) and the European Medicines Agency (EMA) in doses 1.5, 3.0, 4.5, and 6.0 mg/day. In addition, the FDA granted approval for the treatment of adult bipolar I patients in manic, mixed, or depressive episodes as well. Cariprazine is a dopamine D_3_-D_2_ and 5HT_2A_ partial agonist with high affinity and preferential binding to the D_3_ receptors (6). With this unique mechanism of action, cariprazine has the ability to alleviate several symptom domains of the schizophrenia spectrum (7). Indeed, the efficacy of cariprazine in acute schizophrenia was established in 4 Phase II/III, short-term, double-blind and placebo-controlled clinical trials (NCT00404573, NCT00694707, NCT01104779, NCT01104779) (8–11). Furthermore, in a randomized, double-blind clinical trial with an active comparator, Németh and colleagues found support for the notion that cariprazine is effective in the treatment of predominant negative symptoms as well (12).

It is a well-known fact that randomized controlled trials (RCTs) are the gold standard in psychiatric research as they provide the highest quality data regarding the efficacy of a treatment (13). Nonetheless, participants in such trials are highly selected and therefore the “real” patients are often underrepresented (14, 15). In addition, aiming to prove efficacy without inducing undesirable side effects, the doses used in these trials are often lower than what is actually needed in real-life (16, 17). Thus, to understand the effectiveness, i.e., the performance of compounds in everyday practice, it is important to collect data in real-life settings too (18). The aim of this paper is to update the existing evidence on the effectiveness of cariprazine in real-life settings in patient on the schizophrenia spectrum who were treated for only a short time (2 weeks) with high doses (even outside the approved dose range) by presenting data from a Spanish hospital.

## Methods

This was a retrospective real-world data collection conducted in Infanta Sofia University Hospital. All patients provided informed consent to participate in the data collection.

Adult patients between ages 18 to 65 years old who were admitted to the hospital, were diagnosed with disorders on the schizophrenia spectrum (based on DSM-V or ICD-10) and were prescribed cariprazine based on clinical judgement were included retrospectively in the data collection.

Baseline clinical data such as demographics, disorder and treatment history were collected as usual. Effectiveness of cariprazine was measured *via* the Brief Psychiatric Rating Scale (BPRS) (19) and Clinical Global Impression-Severity (CGI-S) scale (20). These scales were applied at hospital admission and at discharge (2 weeks after admission). All patients were discharged after 2 weeks, as after discharge, patients were monitored by an outpatient center and hence data after discharge is not included in the present analysis. The Clinical Global Impression – Improvement scale was also utilized at discharge. In addition, the tolerability of cariprazine was measured by the UKU Side Effect Rating Scale (21) after 1 week of treatment and at discharge from the hospital. The utilized doses of cariprazine were also noted at admission, after 1 week of treatment, and at discharge.

Statistical analysis was conducted using RStudio, version 2022.02.3+492. For demographics, disorder and treatment history, means, standard deviations and percentages were calculated. Given the small sample size, determining the distribution of scores acquired from the BPRS and CGI-S scales was important for choosing the appropriate statistical method. Thus, the Shapiro–Wilk test was performed that showed that the distribution of these variables did not depart significantly from normality. Based on this outcome, a non-parametric test, the Wilcoxon Signed-Rank test was utilized for understanding whether the scores of the scales were significantly different at discharge in comparison to admission. W-statistic, V-statistic, *p*-values and effect sizes are reported for the above-mentioned tests.

## Results

Altogether, 14 patients were included in the data collection, whose demographic, disorder and treatment history characteristics are summarized in Table 1. The mean age of the patients was 45 years and 64% of them was male. In terms of employment status, half of them was unemployed, while 21%–21% was employed or retired. 50% of the admitted patients were single, 43% married and about one third of them lived with their parents, one third with their own family and one third alone.

**Table 1:** Demographic, disorder, and treatment characteristics.

Total patient number	14
Age, mean (SD), years	45.43 (14.65)
**Sex, *n* (%)**	
Men	9 (64.28)
Women	5 (35.71)
**Employment status, *n* (%)**	
Employed	3 (21.43)
Unemployed	7 (50.00)
Student	1 (7.14)
Retired	3 (21.43)
**Marital status, *n* (%)**	
Single	7 (50.00)
Married	6 (42.86)
Divorced	1 (7.42)
**Living circumstances, *n* (%)**	
Parents	4 (28.57)
Family	4 (28.57)
Alone	4 (28.57)
Residence	2 (14.29)
**Diagnosis, *n* (%)**	
Schizophrenia	10 (71.43)
Schizoaffective disorder	4 (28.57)
**Hospital admissions, mean (SD)**	2.86 (3.55)
0	5 (35.71)
1–5	7 (50.00)
5+	2 (14.29)
**Years of illness, mean (SD)**	13.15 (12.20)
0–5	4 (28.57)
5–10	5 (35.71)
10–20	2 (14.29)
20+	2 (14.29)
Unknown	1 (7.42)
**Suicide attempts, *n* (%)**	
Yes	2 (14.29)
No	12 (85.71)
**Smoking status, *n* (%)**	
Yes	8 (57.14)
No	6 (42.86)
**Other drugs, *n* (%)**	
No	9 (64.28)
THC	4 (28.57)
Cocaine	2 (14.29)
Heroine	1 (7.42)
**Previous antipsychotic treatment, *n* (%)**	
No previous antipsychotic treatment	1 (7.42)
Aripiprazole	4 (28.57)
Asenapine	1 (7.42)
Clotiapine	1 (7.42)
Lurasidone	1 (7.42)
Paliperidone	1 (7.42)
Quetiapine	2 (14.29)
Risperidone	3 (21.43)
Olanzapine	5 (35.71)
**Previous other treatment, *n* (%)**	
No previous other treatment	1 (7.42)
Antidepressant	4 (28.57)
Benzodiazepine	10 (71.43)
Mood stabilizer	2 (14.29)
**Reason for switch, *n* (%)**	
Adverse effects	4 (28.57)
Ineffectiveness	7 (50.00)
Non-adherence	2 (14.29)
Other	1 (7.42)
**Side effects (UKU), *n* (%)**	
**Week 1**	
No side effects	10 (71.43)
Akathisia	3 (21.43)
High intensity	1 (7.42)
Low intensity	2 (14.29)
Headache	1 (7.42)
Very low intensity	1 (7.42)
**Discharge**	
No side effects	13 (92.86)
Discontinuation due to side effect	1 (7.42)
**Other treatment at discharge, *n* (%)**	
No other treatment	5 (35.71)
Antidepressant	3 (21.43)
Antipsychotic	2 (14.29)
Benzodiazepine	4 (28.57)
Mood stabilizer	2 (14.29)

Most of the patients were diagnosed with schizophrenia (71%), while the rest had schizoaffective disorder. The average years of illness was around 13 years, with most patients (36%) being ill for 5–10 years. Many of the patients already had several hospital admissions, on average about 3. Some of them also reported previous suicide attempts (14%). While the majority of patients were smokers (57%), other drug use was not reported by majority (64%). Those who used drugs were associated with THC (29%), cocaine (14%) or heroine (7%).

Except one patient, all others reported previous antipsychotic treatment, most of them olanzapine (36%), aripiprazole (29%) or risperidone (21%). Besides antipsychotics, benzodiazepines (71%) and antidepressants (29%) were also prescribed for many. At the current admission, switching of previous antipsychotic medication to cariprazine was decided due to ineffectiveness (50%), adverse effects (29%), or non-adherence (14%).

**Figure 1:**
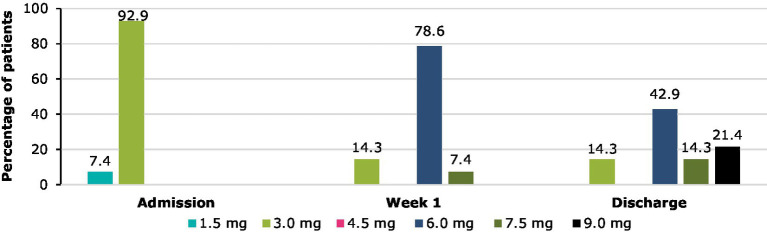
Cariprazine doses between admission and discharge.

93% of patients started cariprazine treatment on 3.0 mg/day and 7% on 1.5 mg/day (Figure 1). After 1 week, the dose was increased to 6.0 mg/day in 79% and 7.5 mg/day in 7% of patients, while 14% stayed on 3.0 mg/day. Fast titration was decided given the severity of symptoms and that the patients were in an inpatient unit. At discharge, the doses were even higher; 21% left the hospital on 9.0 mg/day, 14% on 7.5 mg/day, 43% on 6.0 mg/day and 14% on 3.0 mg/day. High doses were used due to the fact that patients were also on high doses in their previous treatment and safety was closely monitored. One patient discontinued the treatment due to the emergence of severe akathisia. Indeed, 21% of patients reported akathisia after 1 week of treatment according to the UKU (14% only low intensity), however the majority (71%) did not experience any adverse reactions. By the time of discharge, no adverse effects were reported. Besides cariprazine, 29% of patients took benzodiazepines, 21% antidepressants and 14%–14% antipsychotics or mood stabilizers at discharge.

**Figure 2:**
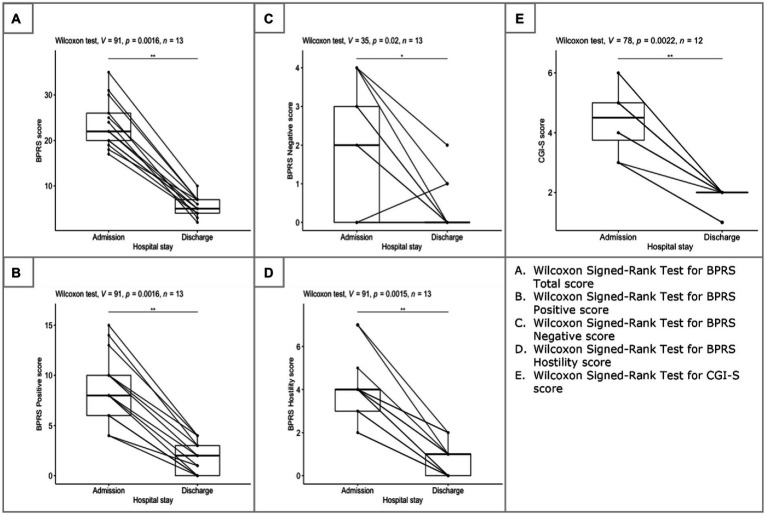
Changes between admission and discharge.

As stated before, the Shapiro–Wilk test was performed to evaluate whether the scores of BPRS and CGI-S are distributed normally. Results of this test are presented in Table 2 alongside with the results of the Wilcoxon Signed-Rank test. At admission, the median score of BPRS was 22 (mean = 23.36), while at discharge it decreased to 5 (mean = 5.54). Indeed, the Wilcoxon Signed-Rank test indicated that the median ranks at discharge were statistically significantly lower than the median ranks at admission (V = 91, *p* < 0.01) with a large effects size (ES) of 0.88 (Figure 2A). When looking at the sub-scores of the BPRS, similar results were acquired. Despite the negative symptom scores (BPRS items 3, 13, 16, and 18) were quite low in the beginning (mean = 2.14, median = 2), they still reduced significantly (V = 35, *p* < 0.05, ES = 0.64; Figure 2C). In terms of the positive symptoms scores (BPRS items 4, 11, 12, and 15), significant difference between admission (mean = 8.86, median = 8) and discharge (mean = 1.85, median = 2) was also detected (V = 91, *p* < 0.01, ES = 0.88; Figure 2B). Since the analyzed population were acute inpatients, hostility (item 10) was decided to be evaluated separately (Figure 2D). Again, difference between admission (mean = 3.86, median = 4) and discharge (mean = 0.77, median = 1) was significant (V = 91, *p* < 0.01, ES = 0.89). The Wilcoxon Signed-Rank test was also significant in case of the CGI-S scores as well (V = 73, *p* < 0.01, ES = 0–89; Figure 2E). At hospital admission patients were moderately-markedly ill (mean = 4.43, median = 4.5), while at discharge they were between normal/borderline ill (mean = 1.83, median = 2). This was reflected in the results of the CGI-I as well, where patients scored between much improved and very much improved.

**Table 2:** Changes between admission and discharge.

Measurement	Admission		Discharge		Shapiro–Wilk test	Wilcoxon signed rank test^*^		
	*Mean, SD*	*Median, IQR*	*Mean, SD*	*Median, IQR*	*W-stat, p*-value	*V-stat*	*ES*	*p*-value
BPRS	23.36, 5.49	22, 6	5.54, 2.11	5, 3	0.92, 0.23	91	0.88	0.002
BPRS negative symptoms	2.14, 2.38	2, 3	0.31, 0.63	0, 0	0.87, 0.06	35	0.64	0.020
BPRS positive symptoms	8.86, 3.42	8, 4	1.85, 1.63	2, 3	0.95, 0.69	91	0.88	0.002
BPRS hostility symptoms	3.86, 1.70	4, 1	0.77, 0.83	1, 1	0.86, 0.06	91	0.89	0.001
CGI-S^**^	4.43, 1.02	4.5, 1.25	1.83, 0.39	2, 0	0.89, 0.13	73	0.89	0.002
CGI-I^**^	-	-	1.92, 0.29	2, 0	-	-	-	-

* *n* = 13, one patient dropped out.

** *n* = 12, missing data on one patient.

## Discussion

Providing novel evidence regarding high-dose antipsychotic therapy with cariprazine as well as repeating the results acquired in the short-term clinical trials with acute schizophrenia patients (8, 10, 11), the present data collection shows that cariprazine has the ability to significantly reduce not only the negative but the positive and hostility symptoms too, as these domains were the most dominant at admission in this patient group. Although dopamine partial agonists are in general considered to be “weak” and therefore avoided in acute settings, it is clear that with the right dosing strategy, adequate treatment response can be achieved.

Indeed, most of the patients started cariprazine treatment on 3.0 mg/day, which is the second lowest dose of cariprazine, and were then up-titrated to 6.0 mg/day or even 7.5 mg/day within a week. Although cariprazine is recommended to be started on 1.5 mg/day according to the Summary of Product Characteristics (SmPC) (22) and this strategy was utilized in all clinical trials, the 3.0 mg/day treatment initiation was reported in many real-life settings such as in an observational study conducted in Latvia (23) as well as in several case studies (24). In addition, the fact that much higher doses, 7.5 mg/day and even 9.0 mg/day were found in this data collection, supports the notion that real-world patients can differ from those included in clinical trials and that these reports add to the overall knowledge regarding the compound. High-dose antipsychotic therapy is defined as *“A total daily dose of a single antipsychotic which exceeds the upper limit stated in the SmPC”* and is associated with increased risks of adverse drug reactions (25). Nonetheless, in the present data collection cariprazine was well-tolerated by the majority of patients as only a few experienced adverse events such as akathisia; most of them in low intensity that resolved on its own. There was one patient however who stopped treatment due to high intensity akathisia. Akathisia is a common side effect of cariprazine; in a post-hoc analysis of 8 short-and long-term clinical trials akathisia was reported in 14.6% (26). In the clinical trials most akathisia was mild/moderate in nature and was managed successfully by rescue medications (26).

These findings are in line with the results of a study by Nakamura et al. where besides the safety and efficacy, the pharmacokinetics of cariprazine were evaluated in different doses (3.0, 6.0, and 9.0 mg/day) (27). According to the results, at week 1 of treatment, mean trough concentration ratios reached over 90% in the 6.0 and 9.0 mg groups (27). In terms of the active metabolites, desmethyl-cariprazine reached over 90% at week 2 of the treatment in the 3.0 and 9.0 mg/day groups, and didesmethyl-cariprazine concentrations peaked at week 4 in the 6.0 and 9.0 mg/day groups (27). Importantly, no patients in the 9.0 mg/day groups experienced any serious adverse events, and the most common adverse event in this group was akathisia (27). In addition, the good tolerability and safety of cariprazine in high doses were confirmed also on long-term studies (28).

It is also important to note that about one third of the patients in this data collection were diagnosed with schizoaffective disorder. Although currently cariprazine is not approved for the treatment of these patients, it is not surprising that they still benefited from and improved a lot with cariprazine. Schizoaffective disorder is by definition a concurrent occurrence of an equal admixture of both schizophrenic and major affective disorder symptoms (1) and cariprazine was found to be effective in the treatment of acute manic, mixed and depressive episodes in bipolar I disorder (29–33) and as add-on treatment in major depressive disorder (34).

In summary, the short-term intervention with cariprazine for only 2 weeks with doses partly outside of the approved dose range resulted in fast improvement of symptoms in patients with schizophrenia and schizoaffective disorder. Therefore, the present results provide support for the notion that cariprazine in high dosages might be a well-tolerated and effective treatment option for acute patients on the schizophrenia spectrum in real-life as well. Further studies with higher patient numbers are required to solidate this claim.

## Data availability statement

The raw data supporting the conclusions of this article will be made available by the authors, without undue reservation.

## Ethics statement

Ethical review and approval was not required for the study on human participants in accordance with the local legislation and institutional requirements. The patients/participants provided their written informed consent to participate in this study.

## Author contributions

LG contributed to conception of the manuscript, conducted the statistical analysis, and wrote the first draft of the manuscript. ER provided scientific support to the manuscript given his experience with cariprazine and real-world studies. All authors contributed to the article and approved the submitted version.

## Conflict of interest

The authors declare that the research was conducted in the absence of any commercial or financial relationships that could be construed as a potential conflict of interest.

## Publisher’s note

All claims expressed in this article are solely those of the authors and do not necessarily represent those of their affiliated organizations, or those of the publisher, the editors and the reviewers. Any product that may be evaluated in this article, or claim that may be made by its manufacturer, is not guaranteed or endorsed by the publisher.
